# Supplementary material for: Impact of the Physical Cellular Microenvironment on the Structure and Function of a Model Hepatocyte Cell Line for Drug Toxicity Applications
Source: Cells. 2023 Oct 5;12(19):2408. doi: 10.3390/cells12192408 (PMC10572302; doi:10.3390/cells12192408)
Supplement: Supplementary file 1 [file cells-12-02408-s001.zip › cells-2592189-supplementary.pdf]

Common Name	Gene ID	Forward Sequence
**Antithrombin**	SERPINC1	CTTGAGGTAAATGAAGAAGGC
**N-cadherin**	CDH2	CTGGAACATATGTGATGACC
**Fibrinogen gamma chain**	FGG	CTGGGACAATGACAATGATAAG
**Vasodilator-stimulated phosphoprotein**	VASP	GGAATTGCAGAAAGTGAAAG
**Transforming growth factor β1**	TGFB1	AACCCACAACGAAATCTATG
**Zyxin**	ZYX	ACTACCACAAGCAGTACG
**SUN1**	SUN1	GTGTTTCTTCTTACCAGGTG
**SUN2**	SUN2	AGCCTTCAGATTCTCTTCAG
**Hepcidin**	HAMP	GTTTTCCCACAACAGACG
**Sulfotransferase 1A1**	SULT1A1	CTTCTATGAAGACATGAAGGAG
**Glutathione S-transferase A1**	GSTA1	AGGTATAGCAGATTTGGGTG
**Albumin**	ALB	AGCCTACCATGAGAATAAGAG
**Hypoxanthine-guanine phosphoribosyltransferase**	HPRT1	ATAAGCCAGACTTTGTTGG
**DNA topoisomerase 1**	TOP1	CAAAGACGAAGAAGGTAGTAG
**Ubiquitin C**	UBC	CGTCACTTGACAATGCAG

Supplementary Table S1: Predesigned KiCqStart® SYBR® Green Primers used in RTqPCR.

Target	Supplier	Product Code	Application	Dilution	Rationale
SUN-1	Abcam	ab124770	Immunofluorescence	1:200	Structural component of the complex that links the nucleus to the cytoskeleton and is implicated in mechanotransduction
SUN-2	Abcam	ab124916	Immunofluorescence	1:200	Structural component of the complex that links the nucleus to the cytoskeleton and is implicated in mechanotransduction
Albumin	Santa Cruz Biotechnologies	sc-271605	Western Blotting	1:500	Synthesized by the liver and is a commonly used biomarker to assess hepatic function
α-Fibrinogen	Santa Cruz Biotechnologies	sc-398806	Western Blotting	1:500	Glycoprotein that constitutes the major component of a blood clot and is synthesized by the liver, therefore can infer information regarding liver functionality
β-actin	Abcam	ab8224	Western Blotting	1:1,000	Widely accepted loading control commonly used in Western Blot validation

Supplementary Table S2: Primary Antibodies

Primary antibodies and their suppliers, used in both immunofluorescence and western blotting. In both cases primary antibodies were incubated overnight at 4 ^{o}C prior to wash steps and addition of secondary antibodies. Rationale supporting their choice and relevance to the study is also detailed above.
